# Salivary Oxytocin Is Negatively Associated With Religious Faith in Japanese Non-Abrahamic People

**DOI:** 10.3389/fpsyg.2021.705781

**Published:** 2021-08-26

**Authors:** Junko Yamada, Yo Nakawake, Qiulu Shou, Kuniyuki Nishina, Masahiro Matsunaga, Haruto Takagishi

**Affiliations:** ^1^Brain Science Institute, Tamagawa University, Machida, Japan; ^2^Center for the Study of Social Cohesion, University of Oxford, Oxford, United Kingdom; ^3^School of Economics & Management, Kochi University of Technology, Kami, Japan; ^4^Graduate School of Brain Sciences, Tamagawa University, Machida, Japan; ^5^Graduate School of Human Sciences, Osaka University, Suita, Japan; ^6^Department of Health and Psychosocial Medicine, Aichi Medical University, School of Medicine, Nagakute, Japan

**Keywords:** spirituality/religiosity, religious faith, salivary oxytocin, non-Abrahamic religion, endogenous hormone

## Abstract

Spirituality and religiosity have a significant impact on one's well-being. Although previous studies have indicated that the neuropeptide hormone oxytocin is associated with spirituality/religiosity, existing findings remain inconsistent. Some studies have reported a positive relationship between oxytocin and spirituality/religiosity, while other studies have reported a negative association. Herein, we examined the association between endogenous oxytocin and spirituality/religiosity in 200 non-Abrahamic Japanese individuals (102 females, mean age ± standard deviation = 41.53 ± 10.46) by measuring the level of salivary oxytocin and spiritual/religious faith. We found that the level of salivary oxytocin was negatively associated with spiritual/religious faith. Individuals with higher levels of salivary oxytocin tend to have more negative spiritual/religious faith compared with those with low oxytocin levels (e.g., “*Spirituality/religiosity makes people passive and clinging*.”). Moreover, this tendency was only significant in individuals who were not interested in a specific religion. The uniqueness of spirituality/religiosity in Japan could help interpret the current findings.

## Introduction

Spirituality and religiosity have a significant impact on psychological well-being. Although some evidence suggested no apparent relationship between spirituality/religiosity and happiness (Argyle and Hills, 2000; Francis et al., 2003), various studies have reported a positive association between spirituality/religiosity and overall well-being (Fry, 2000; Yoon and Lee, 2004; Ellison et al., 2012; Villani et al., 2019; Dunbar, 2021). For example, practicing religion can moderate personal distress and provide emotional support since God can serve as a secure base (Ellison et al., 2012), while congregations provide social and emotional support (Dunbar, 2021). Experimental studies have also shown that people tend to donate to charity organizations of shared religious identity (Hawkins and Nosek, 2012). Moreover, religious faith can prevent moral transgressions (Johnson, 2005), while religious fragmentation of society can reduce the degree of happiness at a national level (Mookerjee and Beron, 2005).

Previous studies have suggested an association between spirituality/religiosity and oxytocin (Kelsch et al., 2013; Holbrook et al., 2015; Van Cappellen et al., 2016; Imamura et al., 2017). Oxytocin, a neuropeptide hormone, plays a central role in social affirmations (Feldman, 2012; Olff et al., 2013 for review) and contributes to developing an attachment with others, such as mother–infant bonds (Nagasawa et al., 2013; Kim et al., 2014) and owner–pet bonds (Romero et al., 2014; Nagasawa et al., 2015). Oxytocin also contributes to establishing social affiliation. Previous studies showed that intranasal oxytocin administration facilitates prosocial behaviors for in-group members (De Dreu et al., 2010, 2012) and benevolence for in-group members but not for out-group members (Ten Velden et al., 2014). Moreover, oxytocin administration also increases in-group conformity: experimental studies have suggested that individuals who are administered oxytocin intranasally tend to adopt the opinion endorsed by in-group members (Stallen et al., 2012; Edelson et al., 2015).

Oxytocin may also contribute to spiritual/religious affirmations, which constitute one of the important bases of social affirmations. An empirical study showed that plasma oxytocin level was positively associated with spirituality and spiritual transformation among patients with HIV/AIDS (Kelsch et al., 2013). Another study, which focused on salivary oxytocin (sOT) levels, suggested that individuals who reported high levels of spirituality showed high levels of sOT (Holbrook et al., 2015). Moreover, intranasal administration of oxytocin facilitates the feeling of connectedness with humankind and boosts positive emotions during meditations (Van Cappellen et al., 2016).

Although many studies have suggested that endogenous oxytocin is positively associated with spirituality/religiosity, a recent study reported a different finding. Imamura and colleagues examined the association between plasma oxytocin levels and spiritual/religious belief, specifically, the belief in life after death (Imamura et al., 2017). The belief in life after death is one of the positive feelings about spirituality/religion, which provides psychological support to individuals and contributes to their mental stability (Krause et al., 2002). They found that serum oxytocin levels and the belief in life after death were negatively correlated; individuals with higher levels of basal oxytocin tend not to believe in an afterlife.

In the current study, we examined the association between endogenous oxytocin and spirituality/religiosity in Japanese individuals. Most of the previous studies reporting a positive association between oxytocin and spirituality/religiosity have been conducted with people who follow Abrahamic traditions. However, Imamura's findings, which showed a negative association between oxytocin and spirituality/religiosity, focused on Japanese who followed non-Abrahamic traditions. Since such differences in religious tradition may underlie the discrepancy in previous findings, we examined whether Imamura et al. (2017)'s findings can conceptually replicate in those who follow non-Abrahamic traditions. Additionally, Imamura et al. (2017) primarily focused on elderly people, while other studies focused on a wider age range (Van Cappellen et al., 2016). Therefore, to examine the robustness of the association between oxytocin and spirituality/religiosity in those who follow non-Abrahamic traditions, this study was conducted among people in their 20s and 50s.

Although most of the previous studies used plasma oxytocin to measure endogenous oxytocin levels, we used the sOT level. sOT has recently gained attention as a useful measurement of the endogenous oxytocin level (Carter et al., 2007; de Jong et al., 2015; Martin et al., 2018). Thus, in this study, we examined the oxytocin concentration in the saliva. The validity of sOT as an index of endogenous oxytocin level shall be discussed in the following materials and method section.

## Materials and Methods

### Participants

In the current study, we analyzed a part of a database from a large research project initiated in May 2012 and continued to the present. Six hundred participants in their 20s and 50s living in the suburbs of Tokyo were registered in the research project, and 564 participants were included in the first wave of the study. An overview of the project is shown in Supplementary Figure 1. All the experimental protocols of this research project were approved by the Ethics Committee of Tamagawa University. All the procedures of the study were conducted following the guidelines as set out in the Declaration of Helsinki. All participants provided written informed consent prior to participating in each experiment of this project.

We analyzed 200 participants who completed a spiritual/religious faith questionnaire and provided their saliva samples (mean age ± standard deviation = 41.53 ± 10.46). Spiritual/religious faith was measured in the seventh wave (*n* = 451, from Oct. 2014 to Jan. 2015), and their saliva sample was collected in the ninth wave (*n* = 290, from Nov. 2016 to Mar. 2018) of the study. Of the 290 participants who took part in the ninth wave, saliva samples were collected from 205 participants. Although there is a discrepancy between the timing of oxytocin collection and the measurement of spiritual/religious faith, previous studies have suggested individual stability of both oxytocin levels (Schneiderman et al., 2012; Feldman et al., 2013; Gordon et al., 2017) and religious faith (Koenig et al., 2008; Koenig, 2015) at least from months to years. Some studies have already reported findings concerning the dataset of this research project (Yamagishi et al., 2014, 2015, 2016, 2017a,b; Nishina et al., 2015, 2018, 2019; Matsumoto et al., 2016); none of the studies examined the association between spirituality/religiosity and sOT levels.

### Spiritual/Religious Faith

Although Imamura et al. (2017) have examined the belief in the afterlife in Japanese individuals, other previous studies measured other aspects of spirituality/religiosity, such as interconnectedness to others (Van Cappellen et al., 2016). Therefore, the current study examined participants' general spiritual/religious faith by using the Spiritual/Religious Faith Scale, which consists of 40 items (Zhang and Takagi, 1989). The scale includes the following six subscales: (1) regarding religion as emotional support, (2) believing in the existence of “*Kami* (God),” (3) viewing religion as interconnectedness, (4) indicating the negative sides of religion, (5) regarding religion as a human weakness, and (6) recognizing the existence of a supreme being. Participants completed the scale by using a 4-point Likert scale (1 = “Strongly disagree,” 4 = “Strongly agree”). The reliability of each subscale is shown in Supplementary Table 1.

This scale also includes a measurement of the involvement in spirituality/religiosity. Participants were asked to answer how they were involved in spiritual/religious congregations by choosing one of the six choices and varying the degree of involvement in spirituality/religiosity (e.g., “I belong to a specific spiritual/religious congregation and firmly believe in it.” and “I'm against any kind of spirituality/religiosity.”). All scale items are shown in Supplementary Table S2.

### Salivary Oxytocin Measurement

A saliva sample was collected from the participants at the beginning of the experiment. The passive drool method was applied using the Saliva Collection Aid (Salimetrics, LLC., Carlsbad, CA). Participants were asked to provide their saliva sample (at least 1.2 mL). Saliva samples collected into cryovials were immediately cooled and stored at −80°C. For each sample, 1 mL of saliva was taken and freeze-dried with FD-1000 (Tokyo Rikakikai Co. Ltd., Tokyo) for approximately 16 h overnight. The freeze-dried saliva samples were dissolved and diluted four times with an assay buffer before the assay. We conducted an enzyme-linked immunosorbent assay with a commercially available oxytocin kit (Enzo Life Sciences, Inc., Farmingdale, NY). The assay was performed in duplicate, and the concentration was calculated using a microplate reader (Sunrise Rainbow RC-R; TECAN Group, Ltd., Zürich) according to relevant standard curves. The intra- and inter-assay coefficients of variation were 6.7% and 8.4%, *respectively*.

The same procedure was applied, as recommended by assay induction methods, except for the extraction step, which uses a novel filtration method to remove interfering proteins to avoid erroneously tagging other molecules as oxytocin. This step would be necessary for a blood sample (McCullough et al., 2013). Previous findings showed that the unextracted plasma oxytocin levels are higher than the extracted ones (Szeto et al., 2011; McCullough et al., 2013). Additionally, there was no significant correlation between oxytocin levels before and after extraction. Conversely, Carter (2014) has argued that the extraction step possibly discards most of the oxytocin, thereby underestimating its levels since oxytocin is filtered by binding to other molecules in the plasma. A recent study demonstrated the methodological validation of ELISA measurement without the oxytocin extraction step by showing the high correlation between extracted and unextracted oxytocin levels in dogs (MacLean et al., 2018). In a previous study, Imamura et al. (2017) also used unextracted oxytocin. Although Carter (2014) has not mentioned about oxytocin in saliva, another study has pointed out that a similar problem may arise with oxytocin in saliva (White-Traut et al., 2009). Furthermore, since the levels of sOT are low (McCullough et al., 2013), the extraction step discards oxytocin as the minimum detection limit of the assay kit even after concentration and induces the high intra-assay coefficient of variation causing unreliable results (Grebe et al., 2017; MacLean et al., 2018). For those reasons, unextracted oxytocin samples have been previously used to measure the sOT level in ELISA assay and examine the relationship between oxytocin level and human sociality (Grebe et al., 2017). Therefore, in the current study, we used unextracted oxytocin samples.

### Statistical Analysis

Results were analyzed using R Version 3.6.3 (R Core Team, 2021). Prior to examining the association between sOT and spiritual/religious faith, we classified the spiritual/religious faith scale into six subscales based on a previous study (Zhang and Takagi, 1989) and calculated the mean score of each subscale. We then conducted a multiple regression analysis with each of the six subscales as the dependent variable, sOT level as the independent variable, and age and sex as the control variables. To avoid multicollinearity, we conducted regression analysis for each subscale separately and interpreted the results using Bonferroni corrected *p*-values.

However, it is complicated to interpret the results for each of the six spiritual/religious subscales separately. Therefore, to simplify the results, a principal component analysis was performed using the mean score of each subscale. Subsequently, we aggregated the subscales of spiritual/religious faith based on the results of the principal component analysis and calculated the mean score of each aggregated item. Subsequently, we conducted a multiple regression analysis with each aggregated item as the dependent variable, sOT level as the independent variable, and age and sex as the control variables.

## Results

### Salivary Oxytocin and Demographic Data

The average level of sOT was 48.65 ± 24.55 pg/mL (mean ± standard deviation). Since the level of sOT distribution was skewed (*W* = 1.554, *p* < 0.001; Supplementary Figure 2), we used log-transformed values in the following analyses. Regression analysis revealed no significant effects of participants' age (β = −0.096, *standard error [SE]* = 0.070, *p* = 0.170) or sex (β = 0.103, *SE* = 0.070, *p* = 0.140) on the sOT level.

### Effects of Salivary Oxytocin Levels on Religious Beliefs

**Figure 1:**
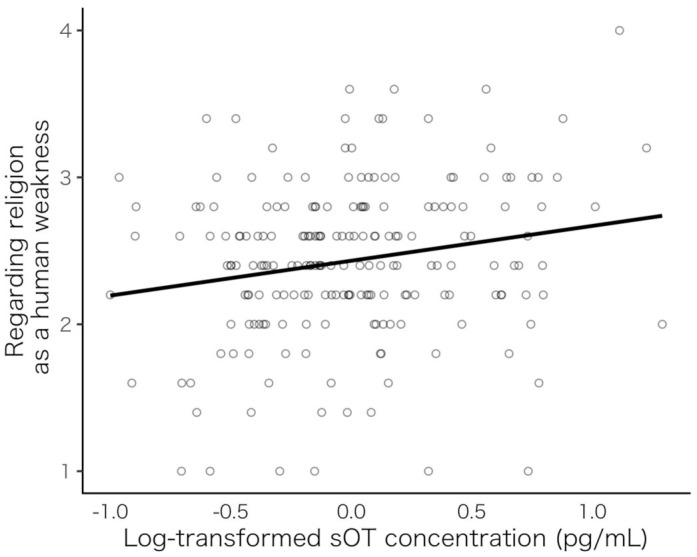
Scatterplot showing sOT levels and faith (age and sex were controlled). Relationship between sOT level and a subscale of religious faith: regarding religion as a human weakness. sOT, salivary oxytocin.

Initially, we conducted a multiple regression analysis to examine the effect of sOT on the six subscales of spiritual/religious faith. The levels of sOT, age, and sex (male = 1, female = 0) were used as independent variables, and each of the six subscales of the religious faith scale was used as a dependent variable. We tested the six subscales of the spiritual/religious faith using Bonferroni corrected *p*-values (*p* = 0.008). A significant effect of sOT was observed on a part of the religious faith subscale: regarding religion as a human weakness (β = 0.194, *SE* = 0.069, *p* = 0.006; Figure 1). Conversely, no significant associations were seen between sOT and other subscales: (1) regarding religion as an emotional support (β = −0.170, *SE* = 0.067, *p* = 0.013), (2) believing in the existence of “*Kami* (God)” (β = −0.142, *SE* = 0.069, *p* = 0.040), and (3) indicating the negative sides of religion (β = 0.158, *SE* = 0.070, *p* = 0.025). Details of the results of each regression model are shown in Supplementary Table 3. The analysis showed that individuals who have higher sOT levels tend to have negative faith in spirituality/religiosity regarding religion as a human weakness.

**Figure 2:**
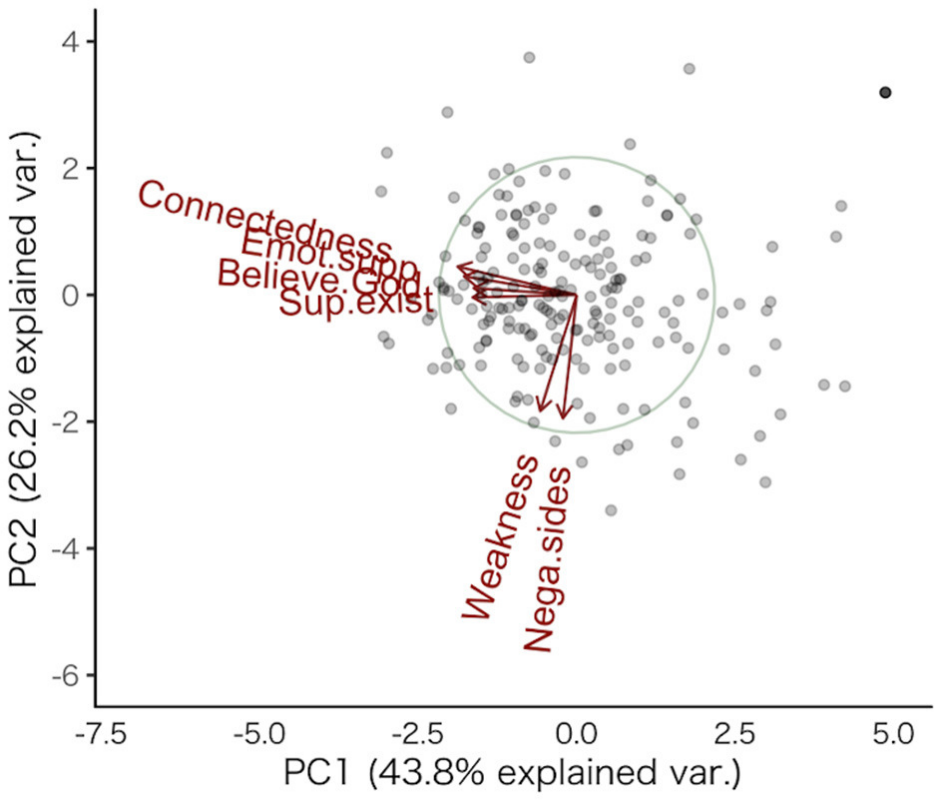
Principal component analysis results. The x-axis indicates PC1, while the y-axis indicates PC2. In total, 43.8% of the variance was explained by PC1, and 26.2% of the variance was explained by PC2. Each red label is an abbreviation of the items (Emot.supp: Regarding religion as emotional support; Believe.God: Believing in the existence of “*Kami* (God);” Connectedness: Viewing religion as interconnectedness; Sup.exist: Recognizing the existence of a supreme being; Weakness: Regarding religion as a human weakness; Nega.sides: Indicating the negative sides of religion). PC, principal component.

**Figure 3:**
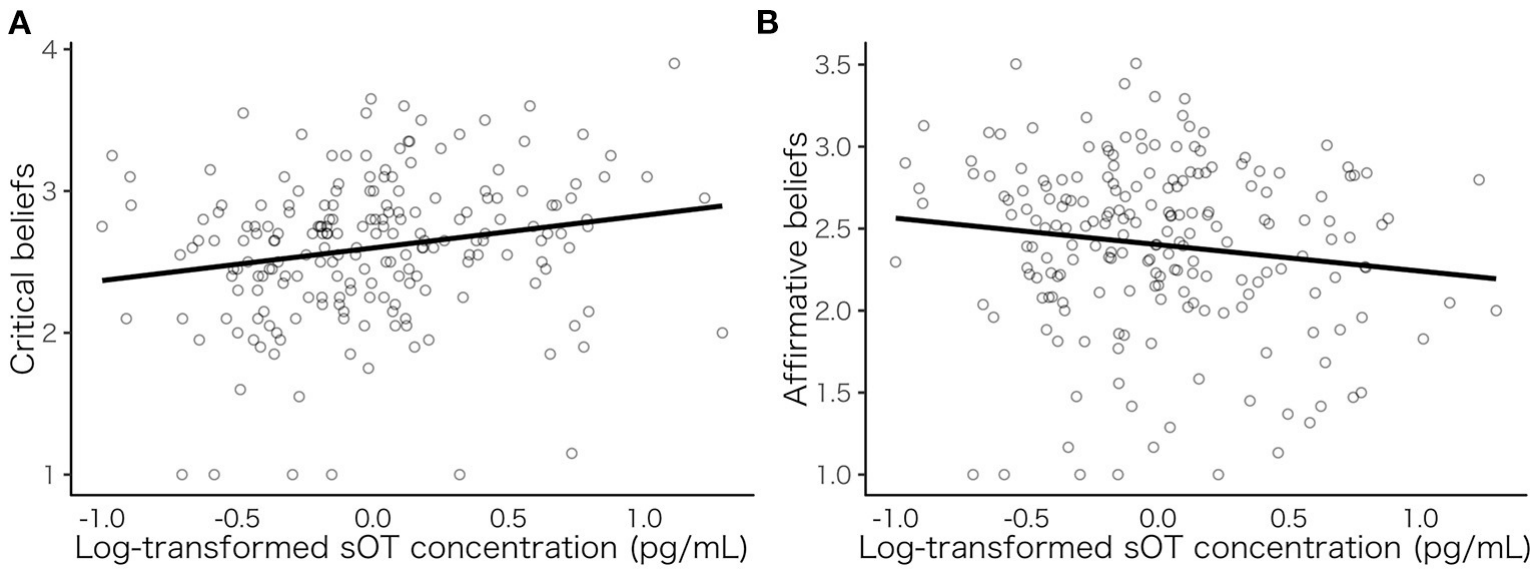
Scatterplots between sOT levels and the two aggregated subscales. Relationship between sOT level and critical beliefs **(A)** and sOT level and affirmative beliefs **(B)**. sOT, salivary oxytocin. Each point represents the average score of each participant.

Thereafter, we conducted a principal component analysis to simplify the preceding results and examine the overall pattern of the sOT levels and spiritual/religious faith (Supplementary Table 4). The cumulative contribution of the first two components was 0.69; thus, several variances could be explained by the two components. We mapped the subscale pattern results as a biplot in Figure 2, which shows two different patterns: the variance of four items is mostly explained by PC1, and the variance of two items is mostly explained by PC2; these items crossed almost orthogonally. Based on these results, we aggregated the subscales into the following two items: “*affirmative beliefs* (in spirituality/religiosity),” which included four items measuring the positive beliefs of spirituality/religiosity (regarding religion as emotional support, believing in the existence of “*Kami* (God),” viewing religion as interconnectedness, and recognizing the existence of a supreme being), and “*critical beliefs* (about spirituality/religiosity),” which included two items related to the negative attitudes or beliefs about spirituality/religiosity (indicating the negative sides of religion and regarding religion as a human weakness). The scatterplot is shown in Figure 3. Subsequently, we conducted a multiple regression analysis with these two new items. The results showed a significant effect of sOT levels on critical beliefs about religion (β = 0.23, *SE* = 0.081, *p* = 0.005, Figure 3A), but not on affirmative beliefs in spirituality/religiosity (β = −0.16, *SE* = 0.081, *p* = 0.045, Figure 3B) at the Bonferroni corrected *p*-value (*p* = 0.025). Individuals with high sOT levels tend to have critical beliefs about religion compared with those with low sOT levels.

### The Moderation Effect of Religious Attitude

Additionally, to examine the moderation effect of spiritual/religious involvement on the association between the sOT and spirituality/religiosity, we divided participants based on their responses on the spiritual/religious involvement scale (Zhang and Takagi, 1989). Participants were divided into the following three categories: high involvement (belonging to a religious organization or believing in a specific supreme being), neutral (not interested in a specific religion), and no involvement (against religion). Since only five individuals fell into the no involvement category, we only analyzed participants in the high involvement and neutral categories. A one-way analysis of variance showed that there was no significant difference in the sOT level between the high involvement and neutral categories [*F*_(2, 193)_ = 0.825, *p* = 0.440].

**Figure 4:**
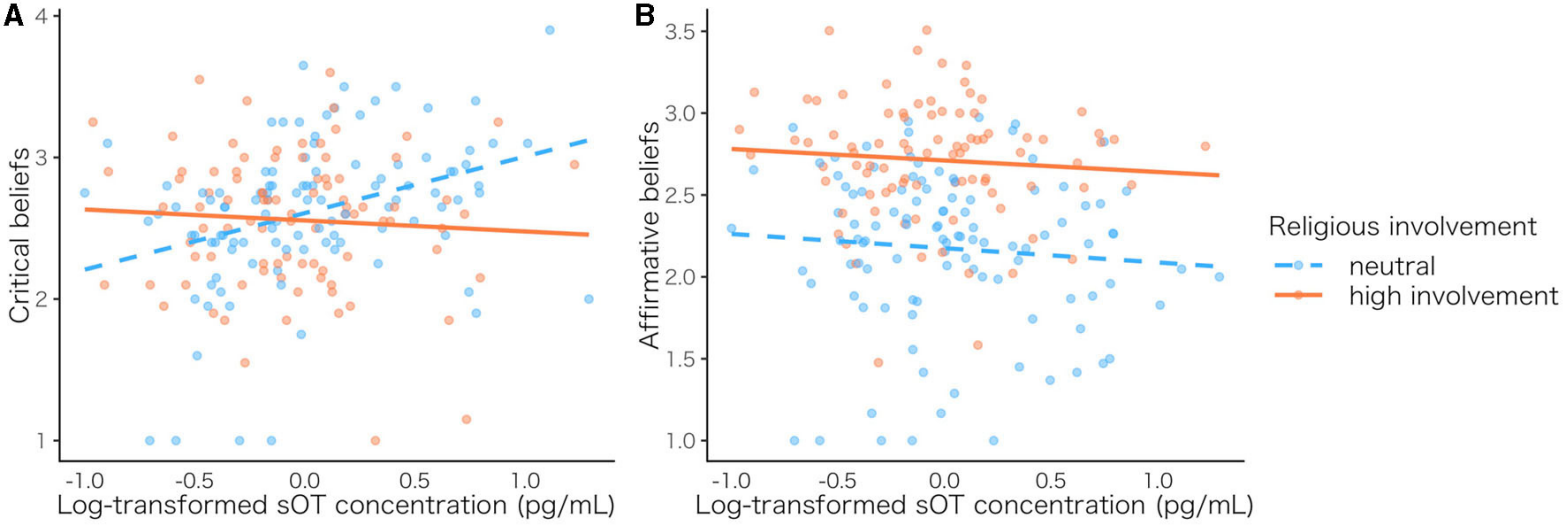
Scatterplots of sOT level and faith for each religious attitude. The orange solid line indicates a positive religious attitude, while the blue dashed line indicates a neutral religious attitude. Relationship between sOT level and critical beliefs **(A)**, and sOT level and affirmative beliefs **(B)**. sOT, salivary oxytocin.

We examined the moderation effect of spiritual/religious involvement using multi regression analysis. The levels of sOT, religious attitude, and the interaction effect (sOT level × religious involvement) were used as independent variables, the religious faith subscales and the new aggregated items were used as dependent variables, and age and sex were used as control variables. There was no significant interaction effect between sOT and religious involvement on each subscale at the Bonferroni corrected *p*-value (*p* = 0.008) (Supplementary Table 5). On the other hand, there was a significant sOT × religious involvement interaction on critical beliefs (β = −0.197, *SE* = 0.070, *p* = 0.005, Figure 4A), but not on affirmative beliefs (β = 0.024, *SE* = 0.059, *p* = 0.687, Figure 4B) at the Bonferroni corrected *p*-value (*p* = 0.025) (Supplementary Table 6). Simple slope analysis revealed that the effect of sOT levels on critical beliefs was only significant in the neutral group (*p* < 0.001). The results indicated that those with high sOT levels tend to have more negative faith toward spirituality/religiosity; however, this pattern was only significant in individuals who were not interested in a specific spirituality and religion.

## Discussion

The current study found significant associations between sOT levels and negative spiritual/religious faith in Japanese individuals. Individuals with high sOT levels tend to have more negative spiritual/religious faith. They tend to perceive religion as a human weakness (e.g., “*Spirituality/religiosity makes people passive and clinging*”) and point out the negative sides of religion (e.g., “*Religion is hypocritical*.”). These results suggested that people with high oxytocin levels would be more suspicious or cautious about believing in religion than those with low levels. This result would be compatible with Imamura's findings, which indicate that those with high levels of basal oxytocin tend to disbelieve in spirituality/religion (Imamura et al., 2017). Interestingly, the association between sOT levels and the critical beliefs about spirituality/religion was significant only in participants who were not interested in spirituality or religion. These results may shed light on the uniqueness of spiritual or religious traditions in Japanese samples, implying that it might be one of the reasons why oxytocin is negatively associated with spirituality/religiosity in the non-Abrahamic samples.

Previous studies showing a positive association between oxytocin and spirituality/religiosity have focused on those who follow the Abrahamic tradition (Kelsch et al., 2013; Holbrook et al., 2015; Van Cappellen et al., 2016). In contrast, Imamura et al. (2017) and the current study, which showed the negative association between oxytocin and spirituality/religiosity, have focused on Japanese individuals who follow the non-Abrahamic tradition. Imamura pointed out that since the majority of Japanese practice a mixture of *Shinto* (indigenous religion in Japan) and Buddhism, the spiritual/religious orientations would be disparate from Abrahamic practitioners (Krause et al., 2002). Based on the *Shinto* tradition, the Japanese can develop an attachment for not only God but also all things in nature including one's ancestors (Imamura et al., 2017).

One possible interpretation of the association between oxytocin and negative spiritual/religious faith is that higher basal oxytocin levels may represent susceptibility to anxiety and stress, and spirituality/religiosity relieves such social distresses. Oxytocin is known as a neural mechanism of social buffering against anxiety and stress (Kikusui et al., 2006; Smith and Wang, 2014). Indeed, oxytocin is secreted in response to social distress (Pierrehumbert et al., 2010; de Jong et al., 2015). In the current study, however, participants' levels of oxytocin were measured as their resting state rather than as a stress response. Previous studies have shown that people who have higher levels of basal oxytocin are more likely to be susceptible to social distress (Tops et al., 2007; Hoge et al., 2008; Oh et al., 2018); those who have higher oxytocin levels could suffer from anxiety and stress. Since spiritual/religious existence (e.g., God) can serve as a haven and relieve individuals from social distress (Ellison et al., 2012), people who have spiritual/religious faith may suffer less from social stress compared with those who have no spiritual/religious faith. Therefore, basal oxytocin levels would be lower in people who have spiritual/religious faith, while they would be higher in those who have no spiritual/religious faith.

Another possible interpretation for the discrepancy between the results of the previous studies and the present study may focus on another function of oxytocin: making individuals more sensitive to out-group threats (Shamay-Tsoory and Abu-Akel, 2016; Egito et al., 2020) and encouraging defensive behaviors such as out-group derogations (De Dreu et al., 2011; Zhang et al., 2019). A recent review argued that oxytocin regulates the salience of social cues such as out-group threats (Shamay-Tsoory and Abu-Akel, 2016). In line with this review, an experimental study showed that individuals who were administered oxytocin were able to judge more accurately the hostility of the out-group members (e.g., armed or non-armed) but not of the in-group members (Egito et al., 2020). Moreover, oxytocin promotes defensive behaviors against out-group threats (De Dreu et al., 2011; Zhang et al., 2019). An experimental study showed that oxytocin administration enhances a negative implicit attitude toward out-group members (De Dreu et al., 2012). Although oxytocin does not directly increase aggression against the out-group, oxytocin promotes coordination within the in-group and enables a more efficient attack on the out-group (Zhang et al., 2019). Since cross-regional studies have shown that Japan is one of the least religious countries (Kavanagh and Jong, 2020) and most of the participants in the current study have no interest in a specific spirituality or religion, spiritual/religious congregations might be perceived as an out-group. Therefore, individuals who have higher levels of oxytocin tend to be more sensitive to the threat of spirituality or religion and negatively evaluate them. In the future, it will be necessary to examine the biological mechanisms of oxytocin's influence on spiritual/religious faith.

### Limitation and Future Directions

First, in the dataset we analyzed, spirituality/religiosity and endogenous oxytocin were measured in the different timestamps. Participants completed their spiritual/religious faith questionnaire in 2015, whereas their saliva samples were collected in 2018. Although previous studies have suggested the individual stability of both peripheral oxytocin levels (Schneiderman et al., 2012; Feldman et al., 2013; Gordon et al., 2017) and religious beliefs (Koenig et al., 2008; Koenig, 2015), some studies argued that oxytocin levels could vary with each day (Martins et al., 2020). Therefore, in future studies, it is required to measure all indexes simultaneously and determine whether the same results are replicated or not.

Second, it would be valuable to measure not only oxytocin but also other types of endogenous hormones simultaneously. The current study focused on oxytocin because it contributes to developing social affirmations including spiritual/religious affirmation (Olff et al., 2013 for review). However, a recent study showed that variation in the β-endorphin, oxytocin, and dopamine receptor genes are differentially associated with human sociality (Pearce et al., 2017). Moreover, previous field experiments suggested that participating in rituals increased the pain threshold, a standard proxy for endorphin activation, and had a positive effect on social bonding (Charles et al., 2020). Thus, exploring the relationship between religion and other endogenous hormones remains to be investigated.

Third, although the validity of measurements of sOT levels adopted in the dataset we used has been demonstrated in a previous study (e.g., Carter et al., 2007; White-Traut et al., 2009; McCullough et al., 2013), there are counterarguments. Some studies suggested that oxytocin levels would differ depending on the measurement method, such as radioimmunoassay and ELISA or some other measuring protocols (Szeto et al., 2011; McCullough et al., 2013). Therefore, taking multiple sOT measurements using different methods could help increase the robustness of the study. Moreover, the current study focused on the basal level of peripheral oxytocin as did the previous study (Holbrook et al., 2015). However, the reliability of the basal level of peripheral oxytocin remains controversial (Valstad et al., 2017). Thus, replication studies with other methodologies (e.g., exogenous oxytocin administration or investing oxytocin receptor gene) are warranted to assess the robustness of our findings.

Finally, another potential future direction is examining the involvement in religious practices, which is related to the religious issues in Japan. Cross-regional surveys often showed that Japan is one of the least religious countries based on a measurement of religious beliefs (Kavanagh and Jong, in press). Consistent with these findings, most of the Japanese participants in the current study were not interested in a specific religion. However, a recent review argued that the measurement of religious beliefs may not reflect religious involvement in Japan, since Japanese religious traditions are more oriented to practices rather than beliefs (Inoue, 2013; Kavanagh and Jong, in press). Thus, measuring participation in religious rituals might be more appropriate to measure religious involvement in Japan, unlike in Abrahamic tradition. The present results could be limited to practice-oriented religious traditions, and oxytocin levels could be positively correlated with belief-oriented religions where sharing religious beliefs could contribute to social identity and bonding. Thus, exploring practice-based measuring and cross-cultural studies, especially compared with a belief-oriented religious tradition, is an interesting direction to explore.

### Conclusion

The current study demonstrated an association between sOT levels and religious faith in Japanese individuals of a wide age range. Individuals with high levels of sOT tend to have more negative faith in spirituality and religiosity. In other words, people who have high sOT levels tend to be more suspicious or cautious about believing in spirituality/religion than those with low sOT levels. These findings were consistent with Imamura's previous findings (Imamura et al., 2017); however, they were inconsistent with most studies examining the association between oxytocin and spirituality/religiosity. The results conceptually replicated Imamura's findings and provided insights into the uniqueness of the spiritual or religious tradition shared by most Japanese individuals. This uniqueness would be useful for interpreting these contradicting findings.

## Data Availability Statement

The original contributions presented in the study are included in the article/Supplementary Material, further inquiries can be directed to the corresponding author/s.

## Ethics Statement

The studies involving human participants were reviewed and approved by Ethics Committee of Tamagawa University. The patients/participants provided their written informed consent to participate in this study.

## Author Contributions

JY, YN, and HT designed the research. KN and HT collected the data. JY analyzed the data. QS, KN, MM, and HT analyzed the oxytocin levels. JY, YN, and QS drafted the manuscript. YN, KN, MM, and HT provided critical revisions. All authors contributed to the article and approved the submitted version.

## Conflict of Interest

The authors declare that the research was conducted in the absence of any commercial or financial relationships that could be construed as a potential conflict of interest.

## Publisher's Note

All claims expressed in this article are solely those of the authors and do not necessarily represent those of their affiliated organizations, or those of the publisher, the editors and the reviewers. Any product that may be evaluated in this article, or claim that may be made by its manufacturer, is not guaranteed or endorsed by the publisher.

## Abbreviations

sOT: salivary oxytocin
SE: standard error.
