# Supplementary material for: Salivary Oxytocin Is Negatively Associated With Religious Faith in Japanese Non-Abrahamic People
Source: Front Psychol. 2021 Aug 26;12:705781. doi: 10.3389/fpsyg.2021.705781 (PMC8427280; doi:10.3389/fpsyg.2021.705781)
Supplement: Supplementary file 1 [file Data_Sheet_1.PDF]

Supplemental Materials

Salivary oxytocin is negatively associated with religious faith in Japanese non-Abrahamic people

Junko Yamada, Yo Nakawake, Qiulu Shou, Kuniyuki Nishina,

Masahiro Matsunaga, Haruto Takagishi

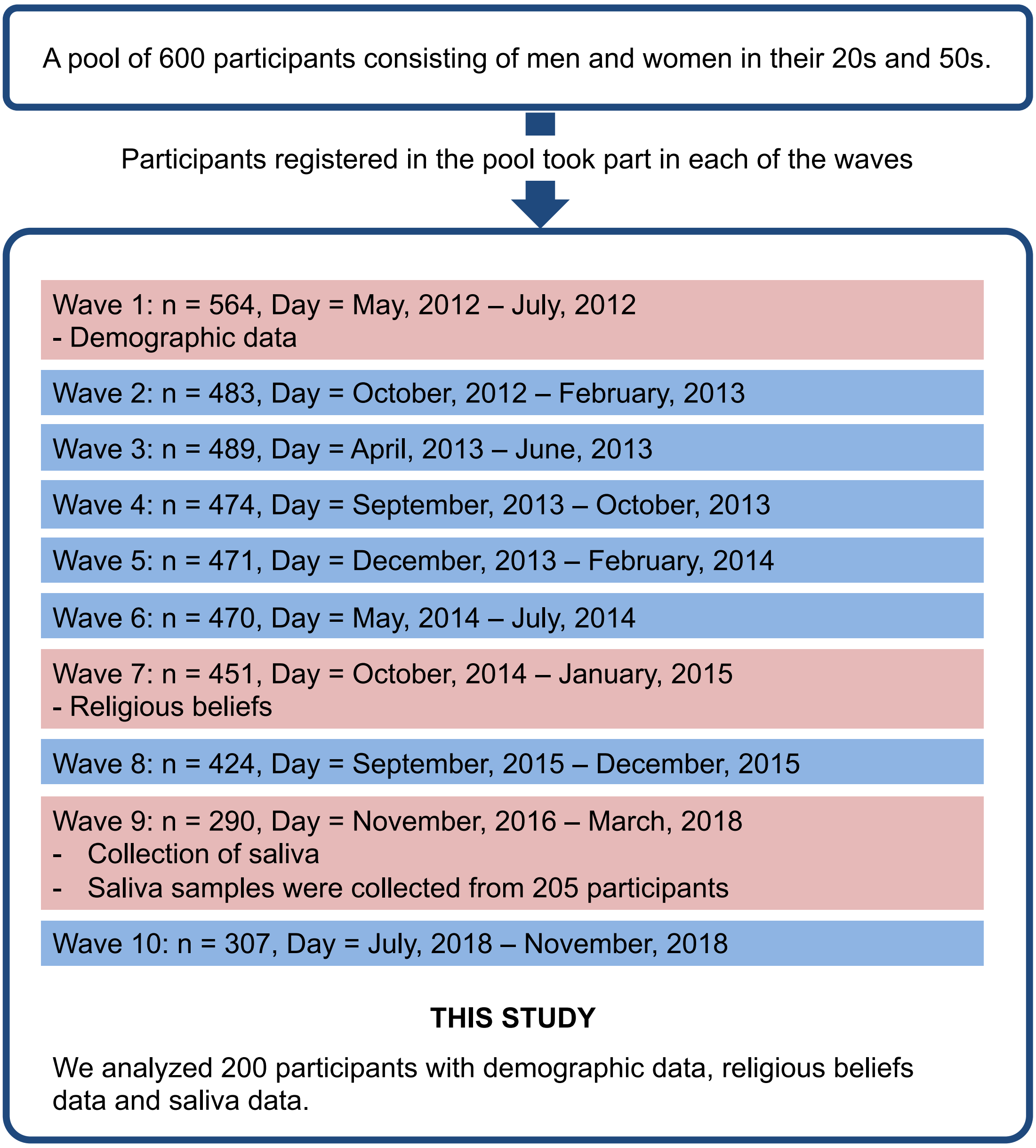

S1 Fig. An overview of the large research project.

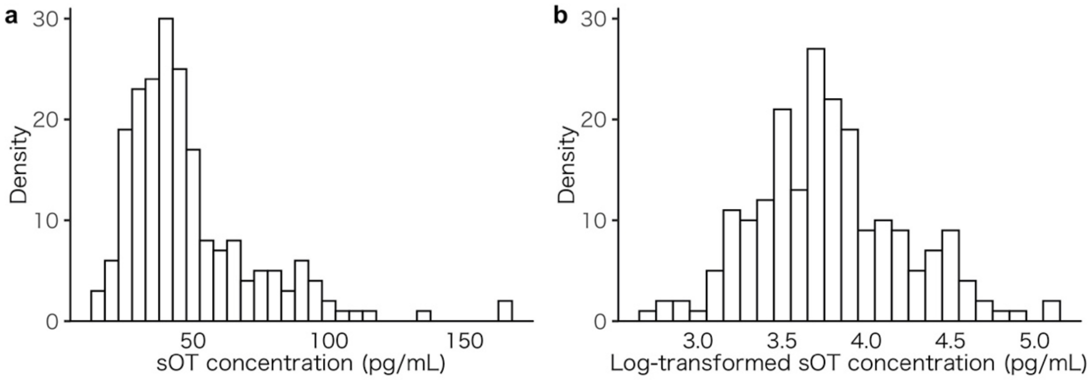

S2 Fig. Salivary oxytocin level histograms. Distribution of salivary oxytocin level before (a) and after (b) log-transformation.

Table S1. The reliability and number of items of subscales of the religious faith and views scale

Subscales	Std. alpha	# of items
Regarding religion as an emotional support	.94	10
Believing in the existence of "*Kami* (God)"	.82	5
Viewing religion as interconnectedness	.92	7
Indicating the negative sides of religion	.93	10
Regarding religion as a human weakness	.72	5
Recognizing the existence of a supreme being	.65	3

Table S2. Religious involvement items. Participants chose one of the items indicated in the table

Items	Category	N
I belong to one religious organization and have strong faith.	positive	3
I belong to an organization or organizations, but I am not active.	positive	15
I do not have faith in established religions, but I have my own faith.	positive	16
I do not conduct activities of religious organization, but I am interested in religions or faith.	positive	55
I do not belong to any religious organizations and do not have interest in religions or faith.	neutral	106
I am against religions.	negative	5

Table S3. Effect of sOT on religious faith (participants' age and sex were controlled).

					95%CI	
Subscales		β	*SE*	*p*	lower	upper
Regarding religion as an emotional support	(Intercept)	-.004	.068	.956	-0.137	0.130
	Age	.228	.068	.001	0.093	0.362
	Gender	-.145	.068	.033	-0.279	0.012
	sOT level	-.170	.067	.013	-0.302	-0.035
	Adjusted R^2	.085				
	F	7.147		<.001		
Believing in the existence of "*kami* (God)"	(Intercept)	-.003	0.069	.968	-0.138	0.133
	Age	.172	0.069	.014	0.035	0.309
	Gender	-.140	0.069	.044	-0.276	-0.004
	sOT level	-.142	0.069	.040	-0.277	-0.007
	R^2	.053				
	F	4.738		.003		

Viewing religion as interconnectedness	(Intercept)	-.005	0.068	.945	-0.138	0.128
	Age	.301	0.068	< .001	0.166	0.435
	Gender	-.065	0.068	.341	-0.198	0.069
	sOT level	-.103	0.067	.129	-0.235	0.030
	R^2	.089				
	F	7.466		< .001		
Indicating the negative sides of religion	(Intercept)	.001	0.070	.990	-0.138	0.140
	Age	.016	0.071	.821	-0.124	0.156
	Gender	-.001	0.071	.899	-0.148	0.130
	sOT level	.158	0.070	.025	0.020	0.297
	R^2	.011				
	F	1.724		.163		
Regarding religion as a human weakness	(Intercept)	.002	0.069	.976	-0.135	0.139

	Age	-.023	0.070	.742	-0.161	0.115
	Gender	-.115	0.070	.101	-0.252	0.023
	sOT level	.194	0.069	.006	0.057	0.330
	R^2	.037				
	F	3.548		.016		
Recognizing the existence of a supreme being	(Intercept)	-.001	0.071	.986	-0.141	0.139
	Age	.072	0.072	.317	-0.069	0.213
	Gender	.006	0.071	.929	-0.134	0.147
	sOT level	-.027	0.071	.704	-0.167	0.113
	R^2	-.009				
	F	0.386		.763		

Abbreviations: sOT, salivary oxytocin; SE, standard error; CI, confidence interval.

Table S4. Results of the principal component analysis (factor loadings).

Subscales	PC1	PC2	PC3	PC4	PC5	PC6
Regarding religion as an emotional support	-.519	-.109	.599	-.055	.135	-.581
Believing in the existence of "*Kami* (God)"	-.472	-.010	-.510	.680	.198	-.121
Viewing religion as interconnectedness	-.498	-.150	.313	.028	.001	.794
Indicating the negative sides of religion	-.038	.782	.062	-.124	.598	.103
Regarding religion as a human weakness	-.111	.590	.169	.266	-.735	-.033
Recognizing the existence of a supreme being	-.495	.082	-.500	-.669	-.213	-.074
Standard Deviation	1.045	0.731	0.540	0.471	0.349	0.302
Proportion of Variance	0.464	0.227	0.124	0.094	0.052	0.039
Cumulative Proportion	0.464	0.691	0.815	0.909	0.961	1.000

Table S5. Moderation effect of religious attitude on the relationship between sOT level and religious faith.

					95%CI	
Subscales		*β*	*SE*	*p*	lower	upper
Regarding religion as an emotional support	(Intercept)	.034	0.063	.586	-0.090	0.158
	Age	.155	0.064	.017	0.028	0.283
	Gender	-0.174	0.064	.001	-0.299	-0.048
	Religious attitude	.370	.065	< .001	0.241	0.499
	sOT level	-.118	.063	.062	-0.242	0.006
	sOT × Religious attitude	.036	.064	.572	-0.090	0.162
	R^2	.225				
	F	10.98		< .001		
Believing in the existence of "*Kami* (God)"	(Intercept)	.008	0.063	.905	-0.117	0.132
	Age	.088	0.065	.174	-0.039	0.216
	Gender	-.195	0.064	.003	-0.320	-0.069
	Religious attitude	.432	0.065	< .001	0.303	0.561

	sOT level	-.085	0.063	.179	-0.209	0.039
	sOT × Religious attitude	.019	0.064	.770	-0.107	0.145
	R^2	.240				
	F	11.94		< .001		
Viewing religion as interconnectedness	(Intercept)	.010	0.057	.864	-0.103	0.123
	Age	.212	0.058	< .001	0.097	0.327
	Gender	-.115	0.057	.047	-0.228	-0.002
	Religious attitude	.516	0.058	< .001	0.401	0.631
	sOT level	-.039	0.057	.492	-0.152	0.074
	sOT × Religious attitude	.119	0.060	.047	0.001	0.236
	R^2	.354				
	F	22.776		< .001		
Indicating the negative sides of religion	(Intercept)	.041	0.058	.473	-0.072	0.155

	Age	.218	0.059	< .001	0.101	0.334
	Gender	-.098	0.058	.095	-0.213	0.017
	Religious attitude	.477	0.060	< .001	0.359	0.595
	sOT level	-.029	0.058	.616	-0.142	0.085
	sOT × Religious attitude	.098	0.058	.096	-0.017	0.213
	R^2	.339				
	F	19.36		< .001		
Regarding religion as a human weakness	(Intercept)	-0.013	0.069	.853	-0.148	0.123
	Age	-0.037	0.070	.597	-0.176	0.102
	Gender	-0.119	0.070	.090	-0.256	0.019
	Religious attitude	-0.017	0.071	.814	-0.157	0.124
	sOT level	0.172	0.069	.013	0.037	0.308
	sOT × Religious attitude	-0.173	0.070	.014	-0.310	-0.035
	R^2	.088				

	F	3.629		.004		
Recognizing the existence of a supreme being	(Intercept)	0.017	0.067	.799	-0.1157481	0.150
	Age	0.006	0.069	.928	-0.1300421	0.143
	Gender	-0.007	0.068	.913	-0.1421343	0.127
	Religious attitude	0.353	0.070	< .001	0.2154167	0.491
	sOT level	0.014	0.067	.835	-0.1188454	0.147
	sOT × Religious attitude	-0.068	0.068	.319	-0.2031409	0.067
	R^2	.133				
	F	5.781		< .001		

Abbreviations: sOT, salivary oxytocin; SE, standard error; CI, confidence interval.

Table S6. Moderation effect of religious attitude on the relationship between sOT level and aggregated religious faith.

					95%CI	
Subscales		*β*	*SE*	*p*	lower	upper
Affirmative beliefs in religion	(Intercept)	0.031	0.059	.598	-0.085	0.146
	Age	0.143	0.060	.018	0.025	0.261
	Gender	-0.148	0.059	.014	-0.264	-0.031
	Religious attitude	0.507	0.061	< .001	0.387	0.627
	sOT level	-0.068	0.059	.245	-0.184	0.047
	sOT × Religious attitude	0.024	0.059	.687	-0.093	0.141
	R^2	.329				
	F	18.54		< .001		
Critical beliefs about religion	(Intercept)	-0.028	0.069	.683	-0.164	0.108
	Age	-0.001	0.071	.986	-0.141	0.138
	Gender	-0.068	0.070	.334	-0.205	0.070
	Religious attitude	-0.041	0.071	.568	-0.182	0.100

sOT level	0.156	0.069	.024	0.021	0.292
sOT × Religious attitude	-0.197	0.070	.005	-0.335	-0.060
R^2	.082				
F	3.358		.006		

Abbreviations: sOT, salivary oxytocin; SE, standard error; CI, confidence interval.
